# Controlling civic engagement of youth spanish muslims: Single representation, generational gap, and gender activism

**DOI:** 10.1007/s11562-022-00481-x

**Published:** 2022-03-10

**Authors:** Cecilia Eseverri-Mayer, Ghufran Khir-Allah

**Affiliations:** 1 UCM-Department Methodology and Theory, Complutense University, Campus de Somosaguas, s/n, 28223 Madrid, Spain; 2 Faculty of Social Sciences, Nebrija University, Calle de Sta. Cruz de Marcenado, 27, 28015 Madrid, Spain

**Keywords:** Islamic Heritage, Spanish Muslims, Inclusive participation, Women Engagement, Religious activism

## Abstract

This paper focuses on Muslim Civil Society structures and, more specifically, on the gap between the organizations from the first (migrant) generation and the new grammars of action of new generations of Spanish Muslims. The originality of this article lies in its power to address three fundamental questions: (1) Are the umbrella organizations silencing the demands of Muslim youth?; (2) How do Muslim youth resist such representative hegemony? and; (3) What specific strategies are Muslim women currently developing to gain representation and involvement in mainstream Spanish society? We find evidence for new grammars of action defying traditional authorities and reject decontextualized, asynchronous Islam. Young women are engaging in *broader* and *inclusive* activism, inspired by religion affiliation in order to reinforce their *external solidarity* and engagement in mainstream political structures. By contrast, young men are calling for a new Muslim leadership to reinforce primary solidarity and concentrate more on earning religious rights. For women, religion is a *vector* to participation and for men, it is a *form* of participation.

**Supplementary Information:**

The online version contains supplementary material available at 10.1007/s11562-022-00481-x.

## Introduction

This paper focuses on Muslim civil society structures as a part of the comparative project [name of the project] conducted in Madrid, Paris and London. Despite efforts at understanding such issues as the institutionalization of Islam in Spanish society (Astor et al., 2017; Planet, 2018); the Muslim struggle for civil rights since the first migrant engagement (Guia, 2014, Álvarez-Miranda 2010); youth activism Téllez, 2008, 2011; Téllez & Madonia 2018; Madonia, 2018; Fernández, 2018) and Muslims’ fight against Islamophobia (Lems et al., 2018; Mijares & Martine 2018; Martine, 2018; Ramírez, 2014; Gil, 2018; Fernández, 2018), the analysis of Muslim activism needs to be approached in a way that adds a new perspective to include the current new dynamics of participation and representation. This new approach must also take into account the ideological conflicts, forms of domination (by means of selective interpretations of Islam) and the resistance and empowerment of both younger generations and women’s groups (by new *aperturiste approaches)* occurring throughout *urban* and *local* Muslim civil society.

What is really happening inside urban Muslim civil society? This paper’s originality lies in its power to address three fundamental questions: 1) Are the umbrella organizations^1^ silencing the demands of young Muslims?; How do Muslim youth resist such representative hegemony?; and What specific strategies are Muslim women currently developing? Answering these questions will help to understand how a discriminated minority group in Spain is gaining recognition and confronting the lack of freedom inside and outside the religious community. Studying the growing importance of religious identities among ethnic minorities (Jacobson, 1997; Mushaben, 2008) is crucial to understanding how *religion* is currently shaping political identity and civic participation (O’Toole & Gale, 2013). In a world of rising anti-Muslim sentiment and extremism (both far right and Islamist), knowing how the politics of civil participation is changing among Muslim communities clearly remains a key issue for enhancing an inclusive coexistence in the West.

During the project research (mentioned above), we have noticed that little attention has been paid to the religious dimension in political identity construction of Spanish Muslim generation. In addition, the gap between old and new generation as much as the differences between Muslim men’s and women’s civil participation were not deeply analyzed. This paper aims at spotting the light on how Spanish Muslims challenge *delegation* and decontextualized Islam. It also covers young Spanish Muslim women engagement in *broader* and *inclusive* activism. We argue that Spanish Muslim youths are contesting the authoritarianism and control exerted by institutionalized umbrella organizations and rejecting decontextualized Islam. Young women are defending inclusive activism and using religion as a *vector (vehicle)* of participation, reinforcing external solidarity, though young men reinforce internal loyalties and understand religion as a *way* of participation.

**Fig. 1:**
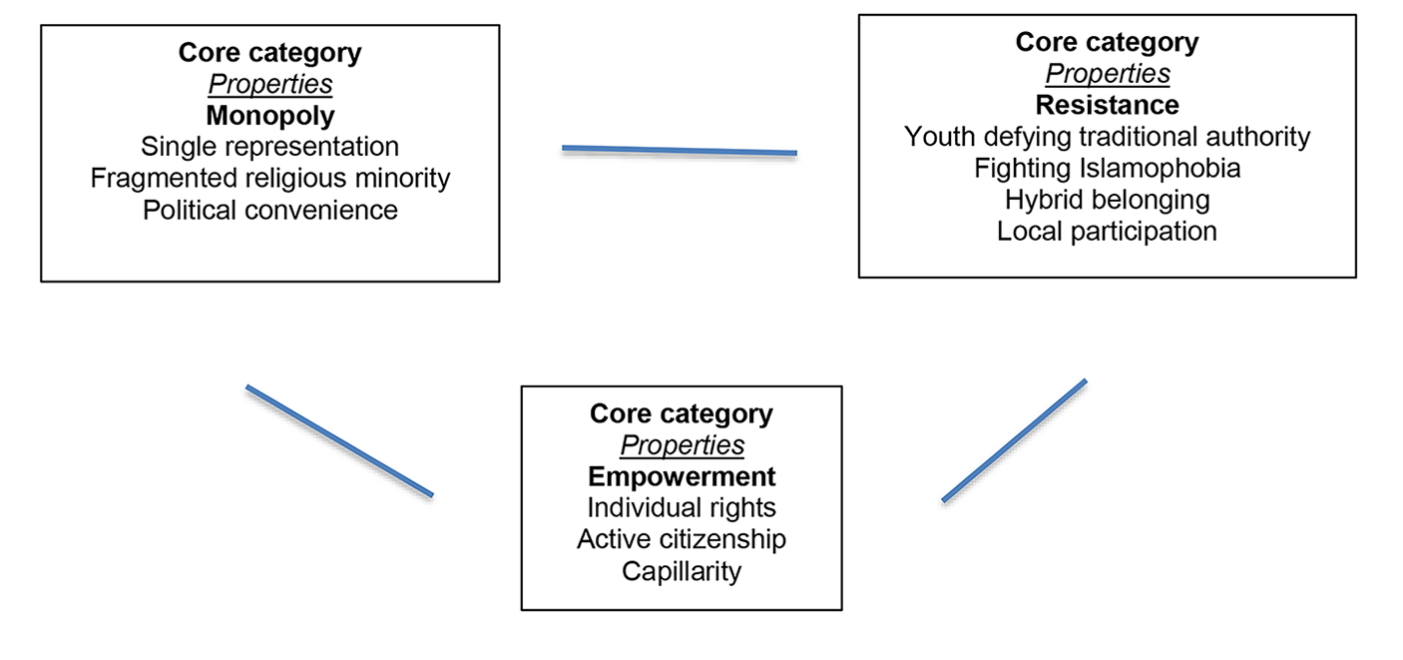
Core categories and properties

The first section of this paper briefly goes through the framework of the Spanish Muslim background, the current legal accommodation of Islamic religion in Spain, and the representative Entities of Spanish Muslim minority. It also goes through international literature about Muslim representation and activism in Europe. In the methodology section, we introduce our participants. To preserve their anonymity, we introduced brief description of their profiles and the Muslim associations we contacted with. We also explain the three core categories of the analysis: monopoly, resistance, and empowerment (see Fig. 1). The analysis section is based on these three categories in the following order: (1) the rule of the game, (2) youth resistance, and (3) women’s self-empowerment. We conclude the paper with a brief summary on the findings along with final reflections.

## Muslim engagement in Spain

Recent studies show that Muslim representation must no longer be regarded as something to be dominated exclusively by Muslim umbrella organizations or the State (O’Toole et al., 2016; Jouili 2015; Kundnani, 2007; Birt 2008; Hammer 2012). Even if the umbrella bodies are perceived from the outside and criticized as unfair “gatekeepers”, notions of what constitutes a “Muslim leader” are being increasingly contested Cesari & McLoughlin, 2005; Joly & Wadia, 2017; Joly, 2017; Lewicki, 2015; O’Toole and Gale 2013; O’Toole et al. 2012). Political representation and new “identity groups” (gender, ethnic, cultural or religious movements) are introducing new forms of political action. Patterns of engagement have changed and “are expressed in more personalized, ad hoc and “DIY” activism” (O’Toole & Gale, 2010; Wieviorka 2005), using information-communication technologies at a “glocal” level (MacDonald, 2006). Young people are skeptical of traditional organizations (political parties, trade unions and associations) and redefine their religious and cultural (instead of ethnic) identity to engage politically and civically (Modood et al., 2006).

Within these changes, Jones et al., (2015: 219) present four new forms of Muslim representation to guide our analysis: (1) *delegation* (represented by umbrella and traditional leaders); (2) *authority* (occurring when there is collaboration between prominent Islamic scholars and the government); (3) *expertise* (an inclusive approach of local participation including women, homosexuals and atheists as expert participants) (O’Toole & Lewicki, 2017) and (4) *standing* (in which influential figures help shape Muslim identity).

Which representative forms might be applicable to urban Muslims in the Spanish capital?

The background of the Spanish Muslim community must be reviewed to address this, starting with the impact of Islam’s historical heritage, or Al-Andalus (the center of Muslim civilization for nearly eight centuries during Islam’s ‘Golden Age’) (Astor, 2014). Spain’s Muslim heritage can still be seen in its culture, language and architecture, despite the Spanish Inquisition’s efforts to eradicate Islam from the Peninsula. “Consequently, some view Spain as a potential bridge between Islam and the West”, explains Astor (2014), a connection rekindled in recent decades due to the high number of immigrants from Morocco, Pakistan, Senegal and Algeria settling mainly in Andalusia, Catalonia, Madrid, Valencia and Murcia.^2^.

This framework has impacted the relationship between Muslims and the Spanish State. Despite the cooperative church–state model developed in recent Spanish history (Díaz-Salazar, 2008)—as a consequence of the Concordat established in 1953 between the Church and the Franco regime (Dietz, 2017)—a new ‘Organic Law of Religious Liberty’ (LOLR) recognizes the cooperation with the State of religious communities which have become ‘deeply rooted’ (notorio arraigo) in Spanish society. Islam was recognized in 1989 as one of the essences of Spanish cultural heritage. A positive step in a period when diversity was not yet an issue of public concern (Astor, 2014; Eseverri-Mayer, 2021, 2021a).

In the first effort to create a legal representative entity that act as intolocutor before the State, Muslim asociations created the *Federación Española de Entidades Religiosas Islámicas* (FEERI) in 18 September 1989, A federation that is co-founded by Masur Escuerdo. During this time, the Muslim Association of Spain (Asociación Musulmana de España- AME), headed by Riay Al-Tatary, and FEERI, headed by Mohamad Bahige, were negotiating the Agreement of Cooperation with the State. AME were not happy with FEERI’s vindictive position during the negotiation especially on the finance issue where FEERI demanded an equalization with the Catholic church. This disagreement, joined to other unsolved issues regarding the systematic methods of representations and public authorities, ended up by creating a new federation: the *Unión de Comunidades Musulmanas de España* (UCIDE), headed by Riay Al-Tarty in 1991. Spanish State required one Muslim representive entity to sign the coopreation, accordingly, both UCIDE and FEERI had to go through unease negociation due to the different positions when demanding the muslim’s community rights. In 1992, both entties formed the *Comision Islamica de España* (CIE), a bicephoalous entity which mantained the heterogentiy of each federation, headed by Riay El-Tatry who maneined a sfoten position at demanding the religous Muslim’s rights^3^.

El Tatry kept his position as a president of CIR, UCIDE and AME until he passed away in April 2020 due to complications of COVID 19. Aiman Adlbi, another Syrian doctor from the first generation of exiled Arabs, was selected as the new president for the CIE and UCIDE. The selection of the president was not an open process for the Muslim community members in Spain. It was limited to CIE board members and it was blessed and encouraged by the Spanish government. The selection (of a similar-background successor) re-opened the leadership-debate in the Spanish Muslim community. Some Muslim leaders criticied that CIE is disregarding non-Arab communities and blocking women and youth participation as decision makers (Cembrero, El Confidencial, 17/07/2020). However, after being selected, Adilbi assigned two Muslim women in CIE board (D. D. Silima Diakite Sylla and Mª Dolores Cuenca). Later on, in Mars 2021, Aiman Adlbi was accused of financing terrorists’ cells and indoctrinate young Muslims. Until today no evidence has confirmed these accusations. Most of Muslim leaders (from the first and second generations) support Adilby against what they consider “false accusations” to the highest representative figure of Spanish Muslim minority. Muslim leaders and activists consider these accusations as a product of the *preventive strategy*, a model that is generalizing suspicion over the Muslim community (Téllez, 2018).

After having a single representation of the Muslim community in Spain in 1992, the CIE signed a Cooperation Agreement with the State to grant the Spanish Muslim community a series of rights (such as the freedom to worship and including Islam in the public education system). Nevertheless, the allocation of only 35 teachers of Islamic education by the Ministry of Education (when the real requirement is 314), the ambiguity on how to regulate the use of hijab in the classroom and workplace, and the exclusion of CIE during the negotiations of the Law against Discrimination in 2019 prove that it has no real worth in practice (Zapata-Barrero and Diez-Nicolás 2012, Khir-Allah 2016, Astor et al., 2017; Álvarez-Miranda 2010). Two main issues stand in the way: (1) the ability of the Islamic Commission to represent the growing young Muslim community in Spain and (2) political and executive-will on both sides: the State and the Islamic Commission, to fulfil the acts in the 1992 Agreement.

International literature identifies a “new grammar of action” in reference to youth Muslim engagement and representation in the West (Jacobson, 1997; Gale and O’Toole 2009; Harris and Roose 2014; O’Toole, 2017; Gest, 2010). In Spain, activism among Muslim youth is also “breaking out” from the invisibility imposed upon them by the single umbrella representation (Téllez and Madonia 2018; Mijares Molina & Martine, 2018). As some ethnographic studies have shown (Eseverri-Mayer 2019; Téllez 2008; 2011; Lems, 2016; Madonia, 2018), Muslim youth and especially Muslim women started appearing in the public sphere after the March 11 attacks (2004), and their visibility was increased after the attacks on Las Rambas (2017), showing that religion is not a barrier but rather a *vector* for full civic and political engagement. A new democratic participation has emerged by Muslim youth, inspired by a “daily and Spanish Islam”, capable of dialoguing with the first generations and with both international Muslim activism and mainstream society (Madonia, 2018). As their claims against Islamophobia were being ignored by the political elite, as Lems has recently demonstrated (2020), they created “New spaces of solidarity and cooperation” with other Europeans associations in order to create an international platform to fight against anti-Muslim sentiment and contribute to the transformation of the world. Other groups are gaining a voice thanks to those who denounce structural and institutional racism within the framework of a decolonial antiracism. This “global” approach reinforces their strategy of action consisting of defining themselves as “Muslims”, “Moors” or “Arabs” to claim their political rights and demonstrating that Spanish citizenship and *Muslimness* are perfectly compatible (Lems, 2020: 248).

However, we have found little attention paid in Spain to the role *religion* is playing in the construction of Muslim political identity, examining difference between generations and genders. International literature has shown the important role young people, and especially women, play in new Muslim activism on issues such as religious rights Rosenberger & Sauer, 2012; Mahmood, 2005; Bano & Kalmbach 2012; Hafez 2011), women’s rights (Lewicki & O’Toole, 2017; Aleksandra & Therese 2017; Mijares & Ramírez 2006; Nyhagen & Halsaa, 2016) and anti-war (Joly & Wadia, 2017; Wadia, 2015) and anti-Islamophobia mobilizations (Brown 2010; Rashid 2014). In Spain, this research is still limited. The fieldwork from this research provides insight on how Spanish Muslims are contesting *delegation* and rejecting a decontextualized and asynchronous Islam. Young women are engaging in *broader* and *inclusive* activism, inspired by religion affiliation in order to reinforce their *external solidarity* and engagement in mainstream political structures to take a stand on non-exclusive and religious issues. By contrast, young men are calling for a new Muslim leadership to reinforce primary solidarity and concentrate more on earning religious rights. For women, religion is a vector to participation and for men, it is a form of participation.

## Methodology

The methodology of this project combined specific ethnographic work through participant observation in different Muslim associations, 25 biographical interviews (Bertaux, 1980) conducted between October 2017 and September 2018 and one focus group with selected leaders (balanced in terms of gender and age) for the purpose of identifying dialogue, tensions and contradictions.

Due to ideological differences and potential conflicts, the names of the associations have been anonymized (Table 1). Few public leaders or policymakers gave us consent to cite them. The sample was composed of 13 women and 12 men between age 18 and 78 (55% of the sample were under 35). Almost all had a university degree, except three (two are still studying and one is a stylist), with a wide variety of professions. Most excelled in their careers as public servants, in the private sector, third sector and in Muslim media or Muslim organizations. Almost half were married with children. All participants define themselves as practicing Muslims, ideologically moderate both in politics and in Islam. 35% feel Muslim, Spanish and European at the same time, though 24% declare themselves feminists, liberal or progressive, in favor of reforming Islam. 20% of the sample defined themselves as “political activists” and the rest feel more comfortable framing their action in a more social, civic and spiritual field.

**Table 1:** Types of associations

*Types of*	*2* *Umbrella organizations*	*2 Principal Mosques*	*5 Youth and civic Association* *(local, student, political, civic and spiritual associations)*	*3 Women Association* *(political, civic and feminist and spiritual associations)*	*2 Global Humanitarian organization* *(actions for war victims, natural catastrophes, poverty and Pro-Palestinian and Syrian associations)*
*First generation*	5	2		1	2
*Second generation*	1	1	4	2	4
*Convert*			1	2	
*Year of foundation*	1981/1989	1988/1992	2017/2004/2004/2008/2018	2009/2004/2009	1991/2004

Accessing the Muslim community, especially leaders, is not easy. Prior ethnographical research by the project’s PI, (anonymized name) with young Muslims in deprived areas in Madrid (Eseverri-Mayer, 2015; 2017; 2019; 2021a) and the engagement of (anonymized name) in the Muslim community facilitated connections to major leaders and activists. Additionally, the possibility of speaking Arabic was key to getting the opinions of elder leaders.

The fieldwork was carried out in three stages: (1) participant observation in various associations (mosques, civil associations and community centers, Arabic language and religious values classes, forums, and debates); (2) 25 biographical interviews and (3) two focus groups with 20 activists and association leaders.

This paper does not claim inductive validity by suggesting that the participants represent the broad experience of Muslims in Spain. Nonetheless, while the sample is limited to 25 cases, the participant observation and biographical interviews allowed for qualitative data to be triangulated (Denzin, 1978) and a better understanding of the place religion holds in their political identity and in the new connections, alliances, tensions and the type of mobilization among first and second Mulsim generations in Madrid. Following intensive analysis and continuous comparison based on Grounded Theory (see Glaser 1992), three core categories of analysis (and its properties) were confirmed: those of *monopoly*, *resistance* and *empowerment* (see Fig. 1).

## The rules of the game. A single (apolitical) representation

The CIE represents Muslims in the Spanish political apparatus (via an advisory commission) but does not exercise political power. The two main leaders of the representative Muslim entities explained they want no part of “political Islam”, demanding religious rights through politics.

*The main objective of the CIE is to monitor cooperation […], if it were political, we would have a (political) representative. It has the aim of Dawa (to preach Islam)” (representative leader, interview, 2018).*

*The federation holds no political stances. A federation is a religious entity, registered in the Ministry of Justice” (representative leader, interview, 2018).*

A common perception among the interviewed Muslim activists indicates that the leader of the CIE –Riay Tatary, for the previous 29 years and now Ayman Adlbi—is named and protected by the State, which blesses this single representation. As such, a considerable range of Muslim associations feel marginalized due to ideological divergences with him:

*There is no political interest in having Muslims engage in real dialogue; and that is the reason they try to keep “someone” at the service of the Administration. You know why? Because if the agreements of ‘92 were actually fulfilled, that means money, because Muslim students, who number over one hundred thousand and have to receive Islamic education, means hiring dozens of teachers. (Religious leader, Interview, Madrid, 2018)*

The fundamental message transmitted by civil society leaders is that this immobility provides both the Spanish State and umbrella institutions positions of comfort: The State has neither implemented nor financed the reforms agreed upon with the Muslim community in 1992, and mainstream Muslim federations neglect to demand it so as to maintain their privileged position. Additionally, the Spanish State wishes to avoid any religious tension that might reveal its privileged relationship with the Catholic Church. Representative Muslim bodies claim they cannot demand too much because of the separation between State and religion. For them, Spanish Muslims have earned guarantees for expressing Islam freely and do not wish to put peaceful coexistence with the other communities in jeopardy. The interlocutor from the CIE indicates that being allowed to practice the Islamic faith in Spain is good enough, other rights are a matter of negotiation:

*“(…) When you are in a country that does not have a Muslim majority… Then one would hardly expect to get pears from an elm tree (…) You have to have a language that is appropriate for the society, proper behavior for living together peacefully (…)I can worship, open mosques, all this is part of Spanish law. And the rest is up for negotiation and discussion.*

Following Jones’s typology, it can be said that the predominant kind of Muslim representation in Spain is based on *delegation*. This type of representation occurs when grassroots associations unify in a federation to put pressure on the State and demand their rights. The problem is that the reputation of these institutional umbrella organizations fails to encourage associations to affiliate. The internal conflict between the two main umbrella federations —UCIDE, run by Syrian refugees, and FEERI, represented by Muslims with a Moroccan background— heightens this perception of misrepresentation. According to the new Spanish Muslim generation, these two entities put too much effort at clashing with each other over public financing. An imam from a Mosque in Madrid explained this tendency:

You know what the problem in Spain is? The problem of Islam in Spain is that most of the people running the mosques, I am very sorry to say, are looking for other things outside of Islam (...). The issue of the federations in Spain is that they are dormant. All they do is hold meetings, meals, [spend] money and [attract] spotlight…

Young people feel underrepresented, viewing these entities as institutionalized, disconnected from real problems that the Muslim community is facing (for example increased Islamophobia, youth extremism, educational challenges at public school, etc.). One engineering student in a youth association in a densely Muslim-populated neighborhood said he is not affiliated to either FEERI or UCIDE because of their passivity, asking for a more vindicative form of engagement.

*(…) They say someone represents us in the community of Madrid and I don’t know who he is! The Griñón problem arose over Muslims who cannot be buried in Madrid, and “the guy” (the President of the CIE) doesn’t show up… I believe that there is a need for the directives to change and for young people to get access!*

A “crisis of trust” in the new young Spanish Muslim generations could be detected among the members from the three representative bodies (FEERI, UCIDE and CIE). These representative bodies questioned and criticized their political and religious orientation and their active role in the civic and political sphere. This fear stems from fundamentalist ideology, political Islam and affiliation to political agendas outside those of these association members (for example those with links to the Moroccan Islamic party “*Justicia y Desarrollo*”). Leaders from FEERI, UCIDE and CIE believe the new generations are still too “unprepared and fragmented” to take the lead and participate in the representative league. For example, a representative member from one entity defends their commitment to Spain’s democratic and secular rules and refers to the other entity’s affiliation to a foreign agenda.

From the start, our project has always meant to be a Spanish Muslim entity, one that does not belong to or have any relation to anyone else. The other project is not defined and in particular had some kinds of foreign relationships, and we have opted not to.

Yet, asking the representative member of the referred-to entity, he negates any political or economic dependence to any foreign party. He said: “No, we defend a model of Spanish Islam and we don’t like having any foreign agenda whatsoever”.

This monopoly of representation sidelines the educated and socially engaged Spanish Muslims’ desire for civil, or even political, participation. Because of these barriers, Muslim youth associations, especially the most active ones, choose to circumvent them and look to alternative forms of representation. For example, the Youth Association of Fuenlabrada (AJUF) preferred not to affiliate with any representative entity. The FAICE (*Federación de Agrupaciones Islámicas por la Convivencia en España*) collaborates with the FIOE (*Federación Islámica de Europa*) while the UCIDE does not. This collaboration is done through the *Liga Islamica por el Diálogo y la Convivencia en España* (LIGOE) whose head offices are in Valencia. Another youth association—Tayba for example—collaborates as well with the *Federación de Pluralismo y Convivencia* which belongs to the Justice Ministry.

## Youth resistance: the new local, urban and anti-islamophobia activism

The demands of Spanish Muslim youth in Madrid are different from those of the previous generation with a migratory background. Their discourse reveals a desire to build a hybrid-space where multi-identities can work together in the public sphere. Interviewees call for a new *multi-hybrid* space of free expression to build Islamic practice based on pure Islam, away from cultural traditions and dogma, and with Spanish cultural elements and characteristics.

*I always say that I grew up somewhere between two realities that in many respects are even contradictory to each other, and I have had to find my way between them. […] I am of Syrian origin, Spanish by birth and Muslim by choice. […] That is why I felt comfortable in a women’s association because I was with girls like me, who felt Spanish and felt Muslim, who did not deny any of their identities.* (21-year-old woman, psychology student affiliated with the student union)

This new capacity for reshaping Muslim identity was already highlighted by some international and Spanish researchers (Téllez, 2011; Madonia, 2018). However, less attention has been paid to analyzing the tensions and conflicts that the youth are experiencing (inside and outside the Muslim community), and the importance of *religion* as a new element of cohesion and mobilization among Spanish Muslims.

One of the first symptoms of these tensions is the reactions of either their parents or Spanish society when expressing this new hybrid identity in public. They are perceived as double agents and untrusted social entities, or as betraying the ethnic community instead of being perceived as rich diverse social entities with multi-cultural values. During the focus group, two women explained:

*If you don’t say you’re Moroccan, it looks like you are denying it. There is a very strong loyalty that you have to demonstrate all the time.*

(Young woman, 26, lawyer and leader of a women’s organization)

*And on the other side you have to explain every day why you are wearing a hijab…and you have to deal with questions about women’s right in Saudi Arabia…It is surreal!*

(Young woman, 22, studying psychology, member of a student union)

Mosques are stagnant places where new complex identities cannot be expressed. Young interviewees demand projects and training courses beyond Arabic and religious values classes, ones that prepare them to be socially and politically active. They believe mosques usually work as mechanisms for containing these changes. They seek to end internal fragmentation and develop a new coordinated activism impacting both the Muslim community internally and local participation of Spanish Muslims in Madrid. In this sense, young interviewees explain that they benefit from the fact that in Madrid (unlike in Catalonia), there is no fixed idea of “nation”, and adaptation is not seen as requiring a loss in ethnic and religious identity Astor, 2012, 2016; Gil Araujo, 2010).

Muslim youth ask for “capillarity” in their actions, which means internal cohesion and the capacity to participate in mainstream civil society moving away from the “ghetto mindset” to reclaim their rights in complex (and even hostile) contexts. Campaigning in the last local election in May 2019, the only female Muslim candidate from the *Ahora Madrid* party began a speech in a meeting with young Muslims stating: “the future of young Muslims is outside the mosques”. Her audience was shocked by this statement, but, as she explained in an interview,

*Mosques do not benefit civic associations in many cases (…) we need more presence, and greater capillarity at the level of coordinated social activism (with broader society); the plenary sessions in city hall and the towns are open once a month for listening in and intervening in this development of the law, but from that perspective. It is not because I am a Muslim that I want something, but rather it is as a citizen that I need the sidewalk to be widened for me*.

(Young 38-year-old woman, PhD-holder in physics and councilwoman of Madrid)

Young Muslim leaders in Madrid place the exercise of citizenship at the heart of their civic and political action, motivated by their beliefs and faith. Islam as a vehicle and not an obstacle:

*Our priority is for them (Muslim youth) to be active citizens. We transmit the values of what moderate Islam is. Like our religion demands that we be open to the outside, not just inside the mosques, because we contribute value through our beliefs: the best thing that Islam has.*

(Young man, 39, leader of a youth group in a Mosque)

A second source of tension between generations and mainstream society is the origins of the imams and their qualifications. Imams in Madrid are Moroccan in most small mosques, Egyptian in the Central mosques, and the imam in The Islamic Saudi Cultural Center is Saudi Arabia. None are Spanish Muslims and they do not speak the language; they are unfamiliar with the cultural differences that would require having Islamic perspectives unlike those from where they came. Some of those imams worked hard to adapt to the Spanish context and learn the language, yet the *Khutbah* on Friday, which is supposed to be the most important communication between the Mosque and the individual, is still in Arabic. Other imams practice only the Islamic school of their countries of origin, even though other Islamic schools might be more adaptable to the real lives of youth in Madrid. In the Central Mosque of Madrid (Abu Baker), Madrid’s second largest and the UCIDE and CIE headquarters, the imam has no effective role at all; his only mission is to lead the prayers and do the Friday *Khutbah* in a language that the youth do not understand. He is from the Sunni Azhar school in Egypt and was hired because his salary is paid by Al-Azhar; the Abu Baker Centre announced that, due to the economic crisis, the Centre cannot afford to pay for an imam.

According to members of youth and women’s associations who were interviewed, this domination of the UCIDE and CIE in the Central Mosque of Madrid—along with the Saudi politically-controlled Islamic Centre, the two major mosques in Madrid— are hampering the adaptation and belonging of the new generation of Spanish Muslims. Most non-official leaders and members of the Muslim community interviewed share a concern for how Muslim youth arbitrarily build their belonging to Spain and the Muslim community, and for how vulnerable the Muslim youth might become in the absence of representative Muslim leaders with whom they can identify. Some association leaders have voiced the need for new female representation, the importance of providing official training for imams in Spain so they might do the *Khutba* in Spanish and communicate effectively with the second generation, and to put forward a common interpretation of the Islamic texts, adapted to the particularities of today’s Spanish and European Muslims. They do not ask for a reform of Islam —“Islam is unique”— but they are *aperturist*, in favor of opening up to new ways of accommodating Islam by “returning to its egalitarian principles” in the Spanish European context.

*I think there is a debate (within the Muslim community), but it is not a public debate, not everyone is being given access. I think there are lots of proposals, but they are proposals from people with power, who have a clear political line and discourse that the rest of the Muslims are not invited to.*

(Young woman, Journalist and Spanish representative of a European Muslim organization)

These demands can be heard from new Spanish Muslims; for example, a 22-year-old woman and active participant who teaches religion in a mosque in Madrid sees a gap between the first generation and second generation on major issues, such as the indications on wearing a hijab:

*(…) once we become aware of this responsibility that we hold and the fact that these children depend on us (…) For example there are children who ask you: “my mother doesn’t wear a hijab” and it turns out that in classes we are telling them that the hijab is obligatory, right? Or that not wearing one is a sin and that she will go to hell. So, we have to be much more careful with that and start with the concerns of the children, (…) They must be able to have a space for themselves.*

Furthermore, the new Spanish Muslims are also critical of mainstream society, pointing out growing Islamophobia in recent years. This phenomenon might be a carry-over from the past and could be used politically by the new Spanish far right political party (VOX). According to the youth, Islamophobia is a battle horse that Muslims must face up to by demanding control and sanctions from political authorities. A vision not reflected in the discourse of the federations that hold power. According to one representative from the Islamic Commission, in Spain “there is no Islamophobia. There is a certain position that arises when there are certain attacks, but the physical attacks in Madrid, Valencia and Navarre after the attacks in Catalonia have been minimal”.

The younger activists do not share the same opinion and carry out ongoing campaigns online to make the victims visible and demonstrate the existence of hateful speech^4^. They have also taken part in the Anti-racism Movement of Madrid, following the example of Black Lives Matters, denouncing the death of Samba Martine (after being ill for 38 days in a CIE-Center of Internment for Foreigners), Mame Mbaye (a peddler who died during police persecution) and Iliass Tahiri (who died after being handcuffed and pinned to the ground by agents for 15 min in a juvenile detention center). They see the danger in the heritage of colonialism and Muslim hate, but at the same time believe in the agency of the new generations.

*’It’s very serious, I believe we have unresolved issues (…) we have to understand that we live in a unique place with a very specific history, we have the whole period of Al-Andalus behind us (…) the evil called the Reconquista, the expulsion… The discourse is being raised and there is an undeniable risk, but at the same time, there is an increasing number of people who both defend diversity and understand Islam in a different way…*.

(42-year-old woman, Islamic feminist and activist against patriarchy and Islamophobia)

The generation of young Spanish Muslims has built a new urban and local space of belonging by resisting traditional authority in Madrid’s main mosques and openly opposing the imposition of certain interpretations of Islam that they consider “atemporal and out of the spatial context”. They want to gain a foothold in the mosques of Madrid, but at the same time are involved at local and the international levels and in the fight against Islamophobia. Activists like the first Muslim councilor woman, youth and women’s associations, Muslim influencers, Instagramers, etc. work with horizontal and local organizations, as well as with globalized networks through new technologies in order to build new online and offline spaces of affiliation and belonging. Paradoxically, religion is getting them outside traditional mosques, letting them stand up to the older generation and draw a path of new political and civic activism.

## Women self-empowerment

Apart from some examples of key women leaders in other Spanish regions^5^ and despite their professional and academic achievements, first and second-generation Muslim women are prevented from accessing leadership positions in Madrid. Only one woman runs an association in Madrid, and that is a young women’s association. Yet, this study reveals that the role of Muslim women is essential in mosques because the Arabic, religious and leisure activities are led by them, mostly as volunteer work. Only one association in Madrid is headed by women.

Due to various obstacles (cultural differences, language deficiencies and integration problems), most first-generation Muslim women never entered the labor market. As a result, they had the opportunity to focus on their qualifications and search for new places to participate in broader society. Their daughters, the new Spanish Muslims, have followed in their footsteps. Consequently, as this anti-Islamophobia activist explains, we see more Muslim women who are better prepared than their male counterparts, thus making them crucial to defining European Islam and leading social change in the public and the political arena.

*We have an imbalance. We have the male youth who are untrained, not ready to live with society (…) The feminine side has studied, has advanced more in their career training and they have a vision of reality that is much more aware than their masculine counterparts’. Then we have the young women who can have the leadership role in social change, redefining Islam or defining European Islam. Men could also fill the need, but they are not so committed…*.

(24-year-old woman, activist at the UCM university and responsible for religious courses at a mosque)

Muslim women face double discrimination, as many researchers have already shown (Mijares & Ramirez, 2016; Joly, 2017; Brown 2010; Abu-Lughod, 2013), both inside and outside the Muslim community. The women interviewed for this study explain that young men usually question the objectives women have growing up in Spanish society. According to them, while these Muslim men are liberal, they also reproduce cultural and traditional traits that affect gender equality. The Muslim city councilwoman in Madrid says that during the election campaign, it was very difficult for her to speak at mosques and cultural associations—“nobody invited me, and I had to make use of other events just to sneak in for 5 minutes and present the *Ahora Madrid* program”. At a mosque in the south of the city where she was allowed to speak, she had to do so with a microphone and in a closed-off adjacent room because the imam refused to let a woman with political discourse be visible during the Friday *Khutba* prayers.

According to these women leaders, the main tool for fighting internal discrimination is to go back to the Islam’s original principals. All the interviewees agree with a gender *ijtihad*, like the one Fatima Mernissi, Margot Badran, Sirin Adlbi Sibai and Amina Wadud defend, and a return to the essence of Islam by recovering the hidden perspective of women’s rights and women’s position in the sacred texts. “It is not actually about taking a step forward and being more modern. The other way around, it is taking a step back and returning to the origins”, says a 23-year-old woman interviewed, with a Law degree and leader of a women’s organization.

For the outsider in the mainstream community, Muslim women are perceived as weak, conditioned and dominated by Muslim men. So here we see a different form of discrimination. This stigmatization ignores the fact that most Muslim women are practicing their religion by choice; they want to be recognized as such and are prepared to engage and work for society (Ramírez & Mijares, 2008). Accordingly, some young Spanish Muslim women tend to hide their religious identity in order to join the workplace easily or resist and confront hijab-discrimination by being consistent on finding job were cultural diversity is respected.

*You confront almost everything. I came across people who denigrate me, saying “if you take off your scarf, then perhaps I can employ you”, and I also have found other companies and tolerant people… Now I am working in a new cabinet and I am very comfortable*.

(Young lawyer and leader of a women’s organization)

Apart from confirming this double discrimination, the originality of this research is to identify that the perspective of young women on social change in the Muslim community is *inclusive* to all Muslim members, regardless of whether they are practitioners or not. Unlike the young male path for social change in the Muslim community, which is *exclusive* and takes very cautious and selective steps. For example, on the controversial topic of homosexuality, Muslim women interviewees tend to include LGBTQ+ Muslims in the Muslim community, separating individual choices from individual rights and duties.

(…) as far as we know, homosexuality has to do with individual practices, it has nothing to do with your behavior within society as long as you comply with your rights and duties.

(25-year-old female activist, member of humanitarian organization, studying Social Work)

Only one male interviewee, an imam in an influential Mosque in Madrid, shared this perspective. He considered homosexuality a sin much like any other that a Muslim might commit in life; and only God judges it. Every other male interviewee, leaders and activists alike, referred to the incompatibility between Islam and homosexuality and the impossibility of collaborating with any political parties that defend individual and civil rights for the LGBTQ+ community. One entrepreneur and association leader from the Sufi community explains: “For example, *Podemos* can be in favor of gay marriage, for example, I can’t commit to that. I am Muslim.” Recently, the Muslim council woman in Madrid and participant in this research officiated a LGBT marriage and showed herself proud of it. She indicated that her action would help stir debate on democracy and citizenship within the Spanish Muslim community. The Muslim community’s reaction varied between those who condemned her act, support her viewpoint, and those who maintained the silence.

The focus group was the technique where the varying views on change and between sexes showed up most. Although both genders recognized the current identity crisis of the young generation, male participants pointed to the lack of an umbrella federation capable of creating a lobby with which put pressure on the State and represent all Muslims in Spain as the cause. They are “on hold” until there exists such representation to demand their religious rights and, only then can they start real social change. Women have a completely different strategy of action. They are aware the Muslim community lacks a clear strategy for civic participation. Yet, they need neither a leader nor a lobby to take action since they have already learned how to participate and become political actors without one. They defend alternative forms of participation and show positive attitudes toward implementing new programs and demonstrating a positive capacity for overcoming challenges. They want to demonstrate to the State that they are capable of contributing to Spanish society, not by making demands for their religious rights, but rather by advancing Spanish society through their qualifications. “Instead of focusing on complaining about Islamophobia or demanding *halal* food in schools, why don’t we show that more and more often we are becoming doctors in public health, that we are presenting environmental projects…?”, says a civil servant and member of a female and international organization.

This research has also found that Muslim women demonstrate broader perspectives on political engagement as well. Male leaders limit political participation to claims-making on the basis of identity and solidarity with the primary ethnic group—ethnicity, religion, costumes or traditions—and are waiting for the moment to achieve it responsibly as a religious minority; by which they become, unconsciously, practitioners of *political Islam*. On the contrary, women, with a conscious understanding of Spanish political patterns, call for a political participation that entails contributing instead of making demands. They ask for a *capillary*, the capacity to move away from the “ghetto mindset”, as a participant defined the concept, and participate in the mainstream society. Despite the fact that Muslim Spanish women suffer intersectional discrimination in daily life that men do not face, they show a deeper understanding of citizenship and a demand-making grounded in forms of broader solidarity. Paradoxically, women show their religious visibility through the *hijab*, yet they want to contribute to the public sphere as autonomous and individual citizens. On the contrary, men do not show any visible religious signs, yet they maintain religious argumentation in political discourse.

Young women call for a new step, a new way forward that includes mixed participation. They see themselves as actors of change and ask: “why do I always have to walk around with my identity as my ID?”, ask one female activist studying psychology. As Islam places the responsibility of financially maintaining the family on men, and due to the very restrictions women face within the Muslim community, Muslim women tend to have more time and volition to be active in secular Spanish civil society than men do. They are engaged in student, humanitarian, feminist and political organizations and achieve positions of leadership. Furthermore, other Muslim women are actively employed and bring a sustainable source of family income despite being mothers. The experience of this first councilwoman proves that the Muslim community is fragmented and far from understanding the game of social change. Direct involvement with wider society or horizontal movements of Muslim women, as other researchers have observed in the UK (O’Toole 2017), are not well supported by male Muslim leaders of Muslim entities. For example, imams and traditional leaders insignificantly supported projects developed by youth associations. They also passively supported the campaign of this first Muslim city councilwoman.

However, even though Muslim women participation and involvement has evolved very positively, they still suffer limitations, as Jones et al., (2015) already noticed when English Muslim women were accused by their community of being “not Muslim enough”; in the Spanish context, Muslim women explain that they are accused of practicing “light Islam”. Young women point out that it is some Muslim women that stand as barriers to their participation because they are socialized to believe they must be “the perfect Muslim woman”, “the housewife” and “the perfect wife and mother”. There is the risk that a self-control mechanism works as a barrier to participation and political involvement in society at large, and only a minority of Muslim women dare take action and become visible public figures. The thoughts of this activist in a humanitarian organization are revealing: “I don’t go to my Muslim friend and tell her my problems or tell her such and such… why? Because as we have it so ingrained in our heads that we have to be perfect, I can’t tell you anything, because it’s scandalous. So, who am I going to tell? To my Spanish friend, who in the end doesn’t mind, but in the end doesn’t understand me. So, I don’t feel like I belong in my world and in the end, I feel alone. What happens? There is that feeling of loneliness that is very scary and you try to feel connected to any group and if you see that in that group, they do welcome you, and what not… and what’s more, you feel useful, you feel like a hero, you feel like you belong to a group, that you’re going to do something good for society…”.

## Conclusions

How religion works as a vehicle for political identity has been a major issue in recent research on Muslim representation (Jones et al., 2015; Lewicki, et al., 2014) and ethnic minority activism (O’Toole, Hammer 2012; Joly, 2015; Wadia 2015). Research being carried out in Spain raises the question as to the place religion holds among new generations (Madonia, 2018). This study seeks to address this question, demonstrating that religion is a vector or even a form of mobilization. It is true that the new generation has not formed a cohesive movement, but instead is developing new more personal, interpersonal, reflexive and informal patterns of political engagement, as literature on ethnic minorities mobilizations has already pointed out (O’Toole & Gale, 2010; Wieviorka 2005). They are adopting new separate initiatives of actions, ones that benefit from the decentralized nature of the participation system in Spain and the “Madrid model”, open to including new demands, sensibilities and identities (Eseverri-Mayer, 2021). For example, they join civic and humanitarian initiatives, they are blogging in favour of a gender-based interpretation of the Qur’an, creating online anti-Islamophobia platforms and demonstrating alongside the Afro-movement against police abuse and racist violence.

They reject limiting themselves to just one association or supporting the umbrella organizations. For them, the umbrella organization is an obstacle to working with young people on preventing radicalization and making progress in active participation. Instead, they want to participate in different initiatives and break down the walls of the Muslim community, engaging in multicultural and multi-ethnic platforms which raise a voice on different issues of social and political life, such as education, coexistence, immigration, housing, youth integration, radicalization… seeking to build bridges with Christians, atheists, and agnostics who share the same projects for society. These isolated and diverse actions show that *religiosity* is the first vector for Muslim people in Madrid when it comes to expressing their concerns about social justice and human rights, though urban and local citizenship is taking the lead in a second phase, especially in the case of women.

This study reveals interesting differences between generations and also genders, which could prove helpful to understanding future dynamics of participation and representation. The first generation of Muslim migrants feels content with being able to practice basic religious requirements in Spain; they do not denounce any form of discrimination and maintain a relationship of convenience with the State, one that guarantees the incurrence of political Islam and prevents radicalization. In contrast, new Spanish Muslims asks for full religious liberty in the Spanish Constitution; they seek to “break down the wall of the mosques” and denounce the increasing Islamophobia while also overcoming fundamentalism by rejecting a decontextualized and asynchronous conception of Islam.

The study also shows the difference between the perspectives of men and women with regard to social change and political action. Where men, unconsciously practicing *political Islam*, are waiting for a Muslim representative leadership to fulfil the ‘92 Co-operation Agreement, women defend a new approach based on *capillarity* and strive to take action in the secular civil and political arenas in order to enact social change through contributions to society as citizens, not as Muslims. This new approach, “Capillarity,” means that they are aware that they have to “negotiate” (and not “dialogue”) with the State. Being prepared, they demonstrate their capacity for creating new civic and political projects that contribute to shaping the identity of the Spanish Muslim community as part of a new model of Muslims, one that is impactful in broader Spanish society. They are aware of challenges arising from culture-traditional interpretations of Islam that might call into question their level of faith (for example with regard to LGBTQ+ community’s rights or defending a gender-oriented reading of Qur’an).

This study concludes that a new generation of women is preparing to be part of the public sphere —as the examples of political women, doctors, engineers, teachers, activists and others demonstrate on a daily basis— and is taking the lead in showing that representation and civic and political participation can be done differently. Young women are showing a *broader* and more *inclusive* activism, drawing inspiration from their religious affiliation to reinforce their *external solidarity* and engagement in mainstream political structures so as to make claims on non-exclusive and religious issues. By contrast, young men are clamoring for a new Muslim leadership to reinforce *primary solidarity* and concentrate more on gaining *religious rights*. For women, religion is a *vector* to participation and for men is *a form* of participation.

Despite these changes and new forms of action and following Jones’ classification of Muslim representation in the UK, we find that the dominant form of Muslim representation in Spain is still *delegation*. Yet, this delegation is limited to one representative figure in Spain, putting the fate of 4% of the Spanish population in his hands under the patronage of the State. This single form of representation might be little more than political cover used to create a self-managed image of Spanish Islam in the European Union. In addition, the State uses this single representation as an instrument to mute the voices of the new generation of Spanish Muslim youth, which is redefining Islam, refreshing feelings of belonging, engaging in Spanish civic life and work on real problems. Such dynamics are showing emerging forms of *expertise* (Jones et al., 2015) in Spanish Muslim civil society. The limitation of these innovative actors is that they are still far from being interlocutors of the Muslim community with the State.

Such a conclusion sheds light on the State’s desire to maintain the comfort zone, by making Spanish Muslim youth invisible through their support of single representation instead of truly improving and supporting the Muslim Youth’s civic and political involvement on an institutional level.

## Electronic supplementary material

Below is the link to the electronic supplementary material.

Supplementary Material 1

## Footnotes

1 An organization that controls and organizes the activities of several other organizations with the purpose of representing Muslims in Spain and interacting with local, regional and national administrations.

2 They represent 4,2% of the total population (1.993.675), of which 42,5% are Spanish Muslims and 57, % foreigners. Among Spanish Muslims, 63,3% are children and grandchildren (UCIDE 2020) and represent 15–19% of youth under 25 years old (Gebhardt et al., 2017).

3 To have a full perspective on the history of the representative entities of the Muslim minority in Spain, read El-Hachim (2021).

4 see: *Plataforma contra la Islamofobia*: https://www.facebook.com/plataformaciuadanacontralaislamofobia. and *Red Musulmanas*: https://www.facebook.com/redmusulmanas/.

5 Such as Carmen del Río (AMDEH (Muslim Association for Human Rights); Isabel Romero (president of *Junta Islámica* and CEO, Halal Institute); Amparo Sánchez Rosell (a Spanish woman and Muslim convert and the only woman to be President of a Muslim Cultural Centre, in Valencia, and founder of the first Citizen Platform against Islamophobia in Spain); Laure Rodríguez (President of *Red Musulmanas*) and Natalia (Ndeye) Andujar (academic and activist).
