# Supplementary material for: Controlling civic engagement of youth spanish muslims: Single representation, generational gap, and gender activism
Source: Cont Islam. 2022 Mar 10;16(1):41–63. doi: 10.1007/s11562-022-00481-x (PMC8907396; doi:10.1007/s11562-022-00481-x)
Supplement: Supplementary file 1 — Supplementary Material 1 [file 11562_2022_481_MOESM1_ESM.docx]

**LIST OF RESPONSES TO THE COMMENTS**

**Title:** "**Controlling Civic Engagement of Youth Spanish Muslims.** **Single representation, Generational gap, and Gender activism**"

This statement outlines the changes conduced to the article in each of the different sections based on the suggestions and corrections made by the two Reviewers. Every change has been written in green.

**Reviewer 2#:**

- As Reviewer #2 has recommended, we have changed the date of the death of Riay Tatary. (P.3)
- We have corrected orthographic mistakes and errors in citations.
- Information about the accusation and arrest of Ayman Adlbi has been introduced in the text, adding the principal reactions of civil society actors from the Muslim community. (P.3)
- We have also cited Lem's (2020) work about Muslim activism. (P.3 and 4)
- We have revised and corrected the list of references.

**Reviewer 3#:**

1. We have followed the recommendations received by Reviewer 3#, and we changed the title. We also have substituted the concept of "unique representation" with "single representation."
2. We have changed the sentence "… the analysis falls short…" and followed the Reviewer 3# (P. 1) recommendations.
3. We have defined "umbrella organization" from the beginning in the introduction of the paper (P.1)
4. We have added the paper argument at the end of the introduction and introduced a brief description of the paper's structure. We also keep our argument at the end of the literature review to clarify our position within the field of research (P.2 and 4).
5. We intended to clarify the background of Muslim representation in Spain, explaining the origins of "ideological tensions" between two factions (one formed of Syrian refugees and the other dominated by Muslims from Morocco). (P.3)
6. In the table about Types of associations, we have added information concerning the foundation year. We could not include the number of members because some of these associations have changed their president and the board committee. They did not want to facilitate our information approved by the previous one. In addition, other associations answered that the number is constantly changing, and they do not have fixed data to be published. (P. 4).
7. We follow the reviewer's recommendation, and we have clearly defined the concept of capillarity and used it later for the analysis (P.8, 12, and 13).
8. We have tried to eliminate some unnecessary repetitions.
9. On p.9, when we talk about "hampering the adaptation, " we refer to the Spanish Muslim youth in general. We are not talking about our sample. This affirmation comes from our interviewees, members, or leaders from non-institutional organizations. Participants in the research have expressed their concerns about feelings of not belonging. New generations of Spanish Muslims are currently suffering (P. 8 and 9).
10. The male leadership is the total number of associations in Madrid. We made it clear in the text. (P.10)
11. We have added more information about the remarkable background of Amparo Sanchez, a Spanish woman and a Muslim convert who was the first woman to run a regional Islamic center (in the city of Valencia) and the founder of the first Citizen Platform Against Islamophobia in Spain (P.10).
12. The ideas expressed in lines 59-60 of the conclusion referred to the fifth type of associations that have been interviewed: "Global Humanitarian organization (actions for war victims, natural catastrophes, poverty, and Pro-Palestinian and Syrian associations). We agreed that in our paper, we had not developed the questions of collective action supporting the Palestinian cause, denouncing the government for collaborating in the Syrian and Yemen war via Islamic. Still, some of these actions are performed by our interviewees. Nonetheless, and following the reviewer's recommendation, we have deleted this part of the text and stressed only the "humanitarian and civic" type of action (P.12).
13. The research and our fieldwork in this project and for this paper have been carried out in the capital of Spain, Madrid. We are currently developing a new project in Ceuta, but it is still in progress, and we have not yet definitive results.
14. We have corrected some style questions: we have explained who "the guy is": *the president of the CIE. We also clarify who are "they": the representative bodies;* Leaders from FEERI, UCIDE, and CIE (P.7).

**Extra note**:

We have corrected some grammatical mistakes in the texts, also marked green.

We have added some "cohesive ties" sentences to introduce the new content in a cohesive consequence.
